# Exercise program design considerations for head and neck cancer survivors

**DOI:** 10.1007/s00405-017-4760-z

**Published:** 2017-10-20

**Authors:** Adrian W. Midgley, Derek Lowe, Andrew R. Levy, Vishal Mepani, Simon N. Rogers

**Affiliations:** 1 Department of Sport & Physical Activity, Edge Hill University, Ormskirk, L39 4QP UK; 2 Evidence-Based Practice Research Centre (EPRC), Edge Hill University, Ormskirk, L39 4QP UK; 3 Department of Psychology, Edge Hill University, Ormskirk, L39 4QP UK; 4 School of Medicine, University of Liverpool, Liverpool, L69 3GE UK; 5 Consultant Regional Maxillofacial Unit, University Hospital Aintree, Liverpool, L9 1AE UK

**Keywords:** Exercise barriers, Exercise benefits, Exercise preferences, Oncology, Physical activity, Rehabilitation

## Abstract

The present study aimed to establish exercise preferences, barriers, and perceived benefits among head and neck cancer survivors, as well as their level of interest in participating in an exercise program. Patients treated for primary squamous cell carcinoma of the head and neck between 2010 and 2014 were identified from the hospital database and sent a postal questionnaire pack to establish exercise preferences, barriers, perceived benefits, current physical activity levels, and quality of life. A postal reminder was sent to non-responders 4 weeks later. The survey comprised 1021 eligible patients of which 437 (43%) responded [74% male, median (interquartile range) age, 66 (60–73) years]. Of the respondents, 30% said ‘Yes’ they would be interested in participating in an exercise program and 34% said ‘Maybe’. The most common exercise preferences were a frequency of three times per week, moderate-intensity, and 15–29 min per bout. The most popular exercise types were walking (68%), flexibility exercises (35%), water activites/swimming (33%), cycling (31%), and weight machines (19%). Home (55%), outdoors (46%) and health club/gym (33%) were the most common preferred choices for where to regularly exercise. Percieved exercise benefits relating to improved physical attributes were commonly cited, whereas potential social and work-related benefits were less well-acknowledged. The most commonly cited exercise barriers were dry mouth or throat (40%), fatigue (37%), shortness of breath (30%), muscle weakness (28%) difficulty swallowing (25%), and shoulder weakness and pain (24%). The present findings inform the design of exercise programs for head and neck cancer survivors.

## Introduction

A mounting body of scientific evidence has shown that physical exercise improves aerobic fitness, strength, physical activity levels, and quality of life, and reduces fatigue in cancer survivors during and post treatment [1–3]. Decreased mortality also has been observed [4]. Accordingly, to promote safe and effective exercise, general cancer and cancer type-specific exercise prescription guidelines have been published [5–7]. An important issue is that sufficient research to support guidelines for less prevalent cancers do not exist and has resulted in the extrapolation of research findings from other cancers [6]. When considering certain cancers often differing in symptoms and treatment strategies, the need for more research to optimise exercise program design for survivors of less prevalent cancers is apparent.

Head and neck cancer represents a diverse set of tumours of the larynx, oropharynx, oral cavity, nasopharynx, nose, and paranasal sinuses [8], with an annual worldwide incidence of over 500,000 [9]. Symptoms consistent with other cancer types include pain, depression, fatigue, and intolerance to physical activity [10, 11]. Other symptoms such as weight loss, head and neck oedema, dry mouth, mouth sores, dysphagia, and shoulder pain and dysfunction are specific to or more dominant in head and neck cancer [10, 12]. Another notable consideration is that individuals presenting with head and neck cancer historically are typically older, with prolonged exposure to smoking tobacco and high levels of alcohol consumption [13]. Comorbidities such as heart and lung disease are therefore typically more prevalent in head and neck cancer survivors [14]. There also has been an increase in younger and fitter individuals presenting with head and neck cancer due to a marked increased prevalence in the human papillomavirus [15]. The cancer-specific symptoms and heterogeneity in head and neck cancer cohorts make the identification of evidence-based exercise guidelines an important challenge for the future.

Only 9% of head and neck cancer survivors have been reported to meet physical activity guidelines after cancer diagnosis [16]. Encouraging exercise uptake and adherence should therefore be an important aspect of their clinical care. Establishing exercise preferences and perceived barriers are important in designing exercise programs that will facilitate uptake and adherence. Although these were previously investigated in the United States [14, 17], the findings were from relatively small samples and might not directly apply to the UK due to cultural and health-care system differences [18, 19]. Another issue is that the percentage of head and neck cancer survivors who regularly engage in exercise is low [16], however, only 17% were reported to feel unable to engage in exercise [14]. Most therefore feel able to engage in regular exercise, but choose not to. An important avenue of enquiry in exploring this issue is establishing the extent to which exercise is perceived as beneficial, since weighing perceived benefits against perceived negative aspects of adopting a behaviour is an important step in deciding whether to adopt that behaviour [20]. Perceived exercise benefits among head and neck cancer survivors have not yet been investigated, however.

The main aim of the present study was to establish exercise preferences, barriers, and perceived benefits among a relatively large sample of head and neck cancer survivors in the UK. A secondary aim was to investigate the level of interest in participating in an exercise program for head and neck cancer survivors, as well as factors associated with between-subject differences in the level of interest.

## Methods

### Participants

University Hospital Aintree is the largest single centre head and neck cancer unit in the UK and in a geographical location with the fourth highest multiple index of deprivation in England [21]. A cohort of patients treated for primary squamous cell carcinoma of the head and neck at the hospital between 2010 and 2014 was identified from the hospital head and neck cancer database. Patients with cutaneous and salivary gland malignancy, patients treated with palliative intent, and patients with recurrence and ongoing disease were excluded. Patients were at least 18 years of age, without known dementia, or other mental condition that could affect their ability to complete the questionnaires used in the study. Mortality status was checked and in February 2016 postal questionnaire packs were sent to all patients known to be alive and disease-free, with reminders sent to non-responders 4 weeks later. Electronic records provided information on clinical characteristics, such as age, gender, year of diagnosis, and treatment. The study received favourable opinion from the Cambridge South NHS Research Ethics Committee (Ref. 15/EE/0429).

### Questionnaire pack

The questionnaire pack contained a covering letter about the survey, instructions on completing the questionnaires, a stamped addressed envelope for return, and the following six questionnaires: (1) exercise preferences; (2) perceived exercise benefits [22]; (3) exercise barriers [17]; (4) Godin leisure time exercise questionnaire [23]; (5) University of Washington quality of life (UW-QoL) Questionnaire version 4 [24]; and (6) ‘Other information’. The Exercise Preferences questionnaire asked whether participants would be *able to* participate in an exercise program for head and neck cancer survivors and whether they would be *interested in* participating. Respondents declaring an interest were asked to answer questions regarding exercise preferences for frequency, intensity, time, and type of exercise, preference for starting the exercise program in relation to the timing of their treatment, and preferred exercise program duration and locations. The perceived exercise benefits questionnaire asked “How do you feel regular physical exercise would/does benefit you?” and provided a list of ten potential benefits previously used in a study involving breast cancer survivors [22]. Each benefit was scored on a Likert scale ranging from 0 (Strongly disagree) to 4 (Strongly agree). The exercise barriers questionnaire asked ‘Regardless of whether you currently exercise, how often do you think the following does/would interfere with your ability to exercise?’ 37 potential barriers were listed, 33 of which were taken from Rogers et al. [17]. The additional four barriers were depression/anxiety, feeding tube, difficulty drinking, and lack of transport. Responses were scored on a Likert scale ranging from 1 (Never) to 5 (Very often). The Godin leisure time exercise questionnaire is a validated measure of self-reported exercise in the community and establishes average weekly frequency of engagement in mild, moderate, and strenuous exercise performed for at least 15 min at a time [23]. The UW-QoL questionnaire data [24, 25] will be reported elsewhere. The ‘other information’ questionnaire asked about age at leaving full-time education, cancer treatment and any recurrence, presence of a feeding tube into the stomach, and co-morbidities.

### Statistical methods

The Chi-squared test was used to compare three groups of respondents (yes interested in participation, maybe interested in participation, not interested in participation) in regard to perceived exercise benefits, exercise barriers, intensity of weekly leisure time exercise, demographic and clinical factors. Statistical significance was accepted as *p* < 0.05. Analyses were performed using SPSS v19 (SPSS Inc., Chicago, IL).

## Results

The survey sample (January 2010–October 2014) comprised 1021 eligible head and neck cancer survivors of which 437 (43%) responded, although 7 of these were omitted from the analyses due to scarcity of questionnaire responses. Lower response was noted for participants aged under 55 years (29%) and over 85 years (36%), but the response was typically 36–50% with no obvious biases when stratified by gender, time from diagnosis, tumour site, squamous cell carcinoma diagnosis, clinical TN staging, treatment group and surgical free-flap status. Median (IQR) time from cancer diagnosis to survey was 43 (30–58) months. The median (IQR) age at survey was 66 (60–73) years and men accounted for 74% (317/430) of respondents. Primary tumours were oral (28%, 122), laryngeal (20%, 86), oro-pharyngeal (41%, 176) and others (11%, 46). The clinical T stage of 27% (113/421) was late (stages 3–4), and the clinical N stage of 39% (164/423) was positive. Primary diagnosis was squamous cell carcinoma for 90% (347/385). Primary treatment comprised surgery alone (41%, 175), surgery with adjuvant radiotherapy/chemotherapy (33%, 143), or primary chemo-radiotherapy alone (26%, 112). Free-flaps were used from almost one quarter (23%, 72/313) of surgical patients and 7% (30/418) of respondents stated they currently had a feeding tube into their stomach. Recurrence of head and neck cancer had occurred in 12% (51/415) of respondents. Two-thirds (67%, 269/399) were 16 years old when they left full-time education.

When asked if interested in participating in an exercise program for head and neck cancer survivors, 64% (267/419) either stated ‘Yes’ (30%, 124) or ‘Maybe’ (34%, 143). Of those with strongest interest, 90% (111/124) stated ‘Yes’ they would be *able* to participate, and 10% stated ‘Maybe’. In those with lesser interest only 30% (43/143) stated ‘Yes’ they would be *able* to participate. One-third of the 267 expressing interest had no preference for exercise frequency, with another third preferring 2 or 3 days per week (Table 1). About half (49%) preferred a program of moderate intensity, with 20% unsure or having no preference. About half (48%) felt *physically able* to exercise for < 30 min, 71% for < 60 min and only 8% for ≥ 60 min, with 21% unsure or unstated. A similar response was observed for *preferred* exercise duration, with those less interested in participating preferring shorter exercising times, with 18% (vs 5% in those with stronger interest) preferring < 15 min. There was little enthusiasm for starting an exercise program before (3%) or during treatment (2%), with more support for starting within a year after treatment (17%) and after 1 year (18%). Most though either had no preference on when to start (30%), or were unsure or did not state (30%). Those more interested in participating felt able to start the exercise program earlier. Preferred program length was ≤ 12 weeks for 26% and > 12 weeks for 26%, and 42% had no preference. Those with less interest in participating preferred shorter programs. The most preferred activities were walking (68%), flexibility exercises (35%), water activities/swimming (33%), cycling (31%), and weight machines (19%). Home (55%), outdoors (46%) and health club/gym (33%) were the most popular choices for where to regularly perform exercise. Those with less interest in participating were more likely to prefer exercising at home (65 vs 44%) than in a health club or gym (21 vs 47%).

**Table 1:** Exercise preferences of those interested in participating in an exercise program for head and neck cancer survivors

	Interested in participating				Total interested (*n* = 267)	
	Yes (*n* = 124)		Maybe (*n* = 143)			
How many days of the week would you like to perform exercise?						
1	7%	9	11%	16	9%	25
2	15%	18	14%	20	14%	38
3	20%	25	20%	28	20%	53
4	8%	10	8%	12	8%	22
5	10%	13	3%	4	6%	17
6	–	0	2%	3	1%	3
7	10%	12	1%	1	5%	13
No preference	30%	37	36%	51	33%	88
Not stated	–	0	6%	8	3%	8
At what intensity would you like to exercise?						
Light	9%	11	25%	36	18%	47
Moderate	52%	65	46%	66	49%	131
Vigorous	17%	21	6%	8	11%	29
No preference	10%	13	6%	8	8%	21
Not sure	8%	10	15%	22	12%	32
Not stated	3%	4	2%	3	3%	7
How long do you think you would be physically able to exercise for?						
< 15 min	12%	15	23%	33	18%	48
15–29 min	31%	38	29%	42	30%	80
30–44 min	19%	24	12%	17	15%	41
45–59 min	10%	12	6%	9	8%	21
≥ 60 min	10%	12	6%	9	8%	21
Not sure	15%	18	21%	30	18%	48
Not stated	4%	5	2%	3	3%	8
How long would you prefer to exercise for?						
< 15 min	5%	6	18%	26	12%	32
15–29 min	36%	45	31%	45	34%	90
30–44 min	17%	21	10%	15	13%	36
45–59 min	13%	16	6%	9	9%	25
≥ 60 min	14%	17	6%	9	10%	26
Not sure	11%	14	22%	31	17%	45
Not stated	4%	5	6%	8	5%	13
When would you feel able to start an exercise program?						
Before treatment	4%	5	2%	3	3%	8
During treatment	2%	3	2%	3	2%	6
0–6 months after treatment	18%	22	5%	7	11%	29
7–12 months after treatment	9%	11	3%	5	6%	16
1 year or more after treatment	17%	21	20%	28	18%	49
No preference	26%	32	33%	47	30%	79
Not sure	15%	18	29%	41	22%	59
Not stated	10%	12	6%	9	8%	21
How long would you like the exercise program to last?						
Less than 6 weeks	9%	11	18%	26	14%	37
7–12 weeks	10%	12	15%	21	12%	33
More than 12 weeks	40%	49	15%	21	26%	70
No preference	37%	46	46%	66	42%	112
Not stated	5%	6	6%	9	6%	15

Stated for	*N* = 120		*N* = 134		*N* = 254	
What type of activities would you like to perform?						
Walking	73%	87	64%	86	68%	173
Flexibility exercises	49%	59	23%	31	35%	90
Water activities/swimming	40%	48	27%	36	33%	84
Cycling	43%	51	21%	28	31%	79
Weight machines	26%	31	12%	16	19%	47
Yoga	20%	24	8%	11	14%	35
Free weights	21%	25	7%	9	13%	34
Resistance bands	18%	22	6%	8	12%	30
Tai Chi	16%	19	7%	10	11%	29
Pilates	14%	17	5%	7	9%	24
Circuit training	16%	19	3%	4	9%	23
Sport	10%	12	5%	7	7%	19
Other^a^	15%	18	7%	9	11%	27
No preference	5%	6	13%	18	9%	24

Stated for	*N* = 119		*N* = 139		*N* = 258	
Where would you like to exercise on a regular basis?						
Home	44%	52	65%	91	55%	143
Outdoors	48%	57	45%	62	46%	119
Health club/gym	47%	56	21%	29	33%	85
Hospital centre	17%	20	7%	10	12%	30
Community centre	15%	18	5%	7	10%	25
Work	2%	2	1%	1	1%	3
Other^b^	4%	5	4%	6	4%	11
No preference	12%	14	9%	13	10%	27

^a^Other activities included (number of respondents in parentheses): treadmill exercise/running (6), rowing (4), golf (4), crown green bowling (3), dancing (3), gardening (3), boxing (1), croquet (1), indoor climbing (1), indoor skiing (1), table tennis (1), trampoline (1), Zumba (1)

^b^Other locations included (number of respondents in parentheses): swimming pool (4), dance school/hall (2), golf course (2), bowling green (1), countryside and coast (1), park (1)

The section on perceived benefits of regular physical exercise (Table 2) was answered by 95% (254/267) of those expressing an interest in participating in an exercise program versus 78% (118/152) of those not interested. The greatest perceived benefits were improving heart and lung fitness (84%), improving health or reducing risk of disease (76%) and building up muscle strength (75%), and lowest for doing better on their job (34%), feeling more attractive (37%) and meeting new people (45%). There was a clear trend for higher perceived benefits from those with more interest in participating. The biggest absolute disparities between those more and less interested were for depression, tension and stress, and self-esteem.

**Table 2:** Perceived exercise benefits of regular physical exercise, for the total sample, and by how interested respondents were in participating in an exercise program

	Agree or strongly agree with statement		Mean score^a^	% Agree or strongly agree			Chi-squared test (3 groups) *p* value
	%	*N*		Yes, interested in participation	Maybe interested in participation	Not interested in participation	
1. Improve my heart and lung fitness	84	301/358	3.1	90	85	77	0.03
2. Improve my health or reduce my risk of disease	76	269/354	2.9	84	77	68	0.02
3. Build up my muscle strength	75	267/354	2.9	91	73	62	< 0.001
4. Lose weight or improve my shape	64	224/348	2.7	77	61	55	0.002
5. Feel less tension and stress	64	226/352	2.7	81	63	49	< 0.001
6. Improve my self-esteem	62	220/354	2.7	79	59	47	< 0.001
7. Feel less depressed	56	200/357	2.6	75	54	39	< 0.001
8. Meet new people	45	160/352	2.4	59	37	40	0.001
9. Feel more attractive	37	130/347	2.2	50	34	28	0.003
10. Do better on my job	34	105/312	2.1	46	29	27	0.005

^a^On a 0–4 scale, with 0 = strongly disagree, 1 = disagree, 2 = neither agree nor disagree, 3 = agree, 4 = strongly agree

The section on barriers to exercise (Table 3) was answered by 98% (261/267) of those expressing an interest in participating in an exercise program and 78% (118/152) of those not interested. The highest rates of scoring as 4 or 5 (very often) on the 5-point barriers scale were for dry mouth or throat (40%), fatigue (37%), shortness of breath (30%), muscle weakness (28%), and difficulty swallowing (25%), with rates for another 13 issues ranging between 20 and 24%. Those not interested in participating were more likely to cite ‘lack of enjoyment’, ‘exercise not a priority’, ‘exercise is boring’ and ‘lack of interest’ as barriers to exercise. They also were less likely to cite ‘lack of equipment’ and ‘lack of facilities and/or space’. Otherwise, the potential barriers were similar regardless of interest in participation.

**Table 3:** Exercise barriers for the total sample and by how interested respondents were in participating in an exercise program

Regardless of whether you currently exercise, how often do you think the following does/would interfere with your ability to exercise?	*N*	Barriers scale				% Scoring 4–5 on the barriers scale			Chi-squared test *p* value
		% 1 = never or 2	% 3	% 4 or 5 = very often	Mean score^a^	Yes, interested in participation	Maybe interested in participation	Not interested in participation	
1. Dry mouth or throat	364	46	14	40	2.9	40	44	38	0.67
2. Fatigue	350	34	29	37	3.0	33	33	42	0.29
3 Shortness of breath	338	53	17	30	2.6	27	35	27	0.31
4. Muscle weakness	338	51	22	28	2.6	25	28	29	0.75
5. Difficulty swallowing	343	62	13	25	2.3	26	26	23	0.85
6. Shoulder weakness and/or pain	337	61	15	24	2.3	29	21	21	0.26
7. Drainage in mouth or throat	315	62	15	23	2.2	24	23	19	0.65
8. Lack of self-discipline	323	50	28	23	2.5	22	22	26	0.73
9. Pain	331	64	13	23	2.2	23	24	21	0.91
10. Difficulty breathing	335	60	18	22	2.3	22	23	20	0.90
11. Lack of facilities and/or space	324	56	22	22	2.4	24	29	9	0.002
12. Difficulty eating	336	64	14	21	2.2	17	24	22	0.46
13. Lack of equipment	319	56	23	21	2.3	24	26	9	0.006
14. Weather	331	51	28	21	2.5	17	25	19	0.29
15. Inconvenient exercise schedule	314	51	29	21	2.5	14	27	20	0.06
16. Exercise not in routine	325	51	28	21	2.5	16	24	24	0.30
17. Exercise not a priority	335	51	29	20	2.5	16	15	29	0.02
18. Lack of enjoyment	337	53	27	20	2.5	15	19	29	0.05
19. Procrastination	279	53	27	19	2.4	22	19	16	0.59
20. Lack of time	323	59	22	19	2.3	19	20	17	0.87
21. Lack of knowledgeable exercise staff	316	64	18	19	2.2	23	20	13	0.16
22. Lack of interest	342	53	29	18	2.4	9	18	26	0.007
23. Exercise is boring	326	58	24	18	2.4	13	18	26	0.04
24. Decreased food intake	326	64	17	18	2.2	15	18	21	0.49
25. Cost	332	66	17	17	2.1	23	17	11	0.09
26. Family responsibilities	328	66	18	16	2.1	15	19	10	0.20
27. Lack of transport	333	76	9	15	1.8	13	16	16	0.70
28. Cough	334	71	15	14	2.0	9	16	16	0.26
29. Fear of making condition worse	329	74	12	14	1.9	9	18	14	0.14
30. Depression/anxiety	326	73	12	14	1.9	10	14	16	0.38
31. Difficulty drinking	330	81	7	12	1.6	11	11	14	0.76
32. Fear of injury	329	76	12	12	1.9	11	14	12	0.77
33. Difficulty communicating	330	78	12	11	1.7	11	11	10	0.94
34. Lack of company	327	75	14	11	1.8	12	14	6	0.14
35. Lack of skills	323	72	16	11	1.9	11	9	13	0.68
36. Feeding tube	321	88	3	10	1.4	12	8	9	0.57
37. Nausea	328	77	13	10	1.7	9	8	11	0.79

^a^On a 1–5 scale, the higher the score the greater the perceived barrier

Further analysis focussed on identifying factors associated with interest in participation. These included current leisure time exercise of > 15 min’ duration one or more times a week, quality of life status, and current clinical and demographic factors. Engagement in strenuous exercise was reported by 12% (51/430), with median (IQR) duration 60 (30–75) min, moderate exercise by 24% (104/428), median (IQR) duration 45 (30–60) min, and mild exercise by 52% (218/417), with median (IQR) duration 40 (30–90) min. One-third (35%, 146/416) did no exercise for > 15 min during their free-time, and of those that did, their median (IQR) weekly leisure activity score was 21 (12–30). Greater current engagement in more intense exercise was associated with greater interest in participating in an exercise program (Table 4), ranging from 52% interest if doing strenuous exercise to 23% if doing no exercise.

**Table 4:** Intensity of weekly leisure time exercise by how interested respondents were in participating in an exercise program

	*N*	Yes, interested in participation		Maybe interested in participation		Not interested in participation		Chi-squared test *p* value
Strenuous exercise^a^ of > 15 min duration one or more times a week	50	52%	26	26%	13	22%	11	0.002
No strenuous exercise but moderate exercise of > 15 min duration one or more times a week	76	38%	29	34%	26	28%	21	
No strenuous or moderate exercise but any mild exercise of > 15 min duration one or more times a week	141	26%	36	35%	50	39%	55	
None of the above	141	23%	32	35%	49	43%	60	
Godin weekly leisure activity score^b^								
0	141	23%	32	35%	49	43%	60	0.007
1–9	60	27%	16	35%	21	38%	23	
10–19	63	33%	21	32%	20	35%	22	
20–29	75	25%	19	40%	30	35%	26	
≥30	68	51%	35	25%	17	24%	16	

^a^Strenuous exercise (heart beats rapidly), e.g., running, jogging, football, squash, roller skating, vigorous swimming, vigorous bicycling. Moderate exercise (not exhausting), e.g., fast walking, tennis, easy bicycling, badminton, easy swimming, dancing. Mild exercise (minimal effort), e.g., easy walking, yoga, fishing, bowling, golf

^b^Weekly leisure activity score (T) calculated from weekly frequencies of strenuous, moderate, and mild activities as follows: T = (9 × Strenuous) + (5 × Moderate) + (3 × mild)

Participant age was a strong indicator of interest in program participation (Table 5), with more than half of those aged > 75 years not being interested. There were no notable associations regarding gender and clinical factors (tumour location, staging, diagnosis & treatment, time from diagnosis) pertaining at the time of primary diagnosis (results not shown). Those whose cancer had returned and those who had chemotherapy showed more interest in participation. Nearly half (48%) stated one or more medical conditions that could impact on their ability to perform exercise and this group had slightly more interest in participating than those not stating any conditions. The three main groups of conditions were joint/mobility-related (92 participants), heart-related (49) and lung-related (46), though for the latter there was less interest in participating.

**Table 5:** Demographic and clinical factors at time of survey by how interested respondents were in participating in an exercise program

	Participants	% Yes, interested in participation	% Maybe interested in participation	% Not interested in participation	Chi-squared test *p* value
Age at survey					
< 55	46	46	37	17	0.002
55–64	140	35	34	31	
65–74	153	25	37	38	
75–79	49	24	22	53	
≥ 80	31	13	35	52	
Age at leaving full-time education					
16	261	29	33	38	0.40
17–18	54	24	44	31	
19–22	35	40	29	31	
Older than 22	40	38	35	28	
Has head and neck cancer ever recurred (ever come back)					
Yes	48	42	38	21	0.04
No	356	28	34	38	
Ever had surgery as part of cancer treatment					
Yes	315	30	36	35	0.54
No	94	29	31	40	
Ever had radiotherapy as part of cancer treatment					
Yes	283	30	35	35	0.72
No	122	27	34	39	
Ever had chemotherapy as part of cancer treatment					
Yes	112	38	33	29	0.03
No	293	26	35	39	
Do you have a feeding tube into your stomach at the moment					
Yes	28	43	25	32	0.23
No	379	28	35	37	
What other medical conditions do you have that could impact on you being able to perform exercise					
Condition(s) stated	199	31	38	31	0.12
None stated	219	28	31	41	
Heart related: e.g., IHD, attack, BP, AF, angina	49	35	41	24	0.20 versus condition not stated
Lung related: e.g. COPD, asthma, SOB	46	17	54	28	0.008 versus condition not stated
Joint/mobility related: e.g., arthritis, hip/knee replacement, osteoporosis, mobility or balance issues, sciatica	92	32	32	37	0.82 versus condition not stated

## Discussion

The main aim of the present study was to establish exercise preferences, barriers, and perceived benefits among a relatively large sample of head and neck cancer survivors in the UK. A secondary aim was to investigate the level of interest in participating in an exercise program for head and neck cancer survivors, as well as the factors associated with between-subject differences in level of interest. Main findings were that 64% of respondents expressed an interest in participating in an exercise program, with greater interest associated with younger age, lower social-emotional aspects of quality of life, absence of lung-related co-morbidity, greater current levels of physical activity, greater perceived exercise benefits, and lower scores on certain barriers to exercise. Exercise preferences were diverse; however, the most popular were a frequency of three times per week, moderate-intensity, 15–29 min per bout, and consisting of walking, swimming, cycling, and flexibility and resistance training exercises. The most commonly cited exercise barriers were symptoms specific to head and neck cancer.

The 30% of respondents in the present study interested and 34% maybe interested in participating in an exercise program were similar to the 33 and 38%, respectively, reported by Rogers et al. [14] for 90 head and neck cancer survivors in the USA. Encouragingly, the most popular exercise program preferences were relatively consistent with current cancer physical activity guidelines [5–7]. A notable exception is that a higher frequency and/or duration would be needed to accumulate the recommended minimum of 150 min of moderate intensity ‘aerobic’ exercise and resistance training on at least 2 days per week [6, 7]. The most popular exercise preferences are useful for designing group-based exercise programs delivered in the community, which dominate the current 312 registered UK cardiac rehabilitation programs [26]. Exercise preferences in the present study were diverse, however, particularly between those more versus less interested in participating in an exercise program. These results emphasise the importance of individuality as a fitness training principle [27] when designing exercise programs for head and neck cancer survivors. Of note is that 55% of interested respondents preferred to exercise at home, with relatively few preferring to exercise in a community (10%) or hospital (12%) centre. This is consistent with a previous observation that 82% of head and neck cancer survivors who showed a preference, favoured unsupervised rather than supervised exercise [14]. The UK cardiac model of group-based exercise programs delivered in a community setting, with home-based programs accounting for only 10% of the total [26], may not therefore be an effective strategy for exercise program design in head and neck cancer survivors. Further support for home-based programs is that no differences in outcomes have been observed between home-based and centre-based exercise programs [28]. There has been a growing interest in telehealth for the remote delivery of exercise programs in clinical populations (e.g., [29]). This may be a particularly effective strategy for delivering home-based exercise programs in head and neck cancer survivors in the UK, given the large distances many head and neck cancer survivors would need to travel to attend their nearest rehabilitation centre.

The most commonly cited exercise barriers in the present study were dry mouth or throat, fatigue, shortness of breath, muscle weakness, difficulty swallowing, and shoulder weakness and pain, which are all dominant symptoms associated with head and neck cancer [10, 12]. These findings are largely consistent with those of Rogers et al. [16], but contrast with those reported for the general adult population, where lack of time and motivation dominate (e.g. [30]). Given the considerable benefits that head and neck cancer survivors can expect from exercise engagement [31], these findings emphasise the importance of providing advice on how to negate or manage disease-specific exercise barriers during standard clinical care. Dry mouth/throat was the most common barrier to exercise expressed by participants in the present study and caregivers should be particularly mindful of the management of these [32], as well as providing advice regarding avoidance of exercise in cold air to prevent exacerbating symptoms [33].

Little research has investigated the perceived benefits of exercise in cancer survivors and, to the best of our knowledge, none has investigated perceived benefits amongst head and neck cancer survivors. The mean values for perceived exercise benefits observed in the present study were lower on all ten items than reported by Spector et al. [22] for breast cancer survivors. The mean values for doing better on my job, improving body shape, feeling more attractive, and meeting new people were the lowest and represented the greatest negative difference compared to those reported by Spector et al. [22]. This might reflect gender differences in perceptions, since 74% of respondents in the present study were men compared to all women in the study by Spector et al. [22]. In the present study, level of interest in participating in an exercise program was positively associated with perceived benefits. This is consistent with the transtheoretical model of behaviour change, which postulates that perceived benefits are important in favourably modifying the decisional balance of the relative weighing of the positive and negative aspects of changing [20]. Of note is that only 13% of participants interested in participating in an exercise program, who also expressed a preference of when they would have felt able to start, felt able to start the program before or during cancer treatment. This is despite mounting evidence of the physical and psychological benefits of exercise before [34] and during treatment [1, 3]. These findings suggest that educating head and neck cancer survivors on the potential benefits of exercise should be an integral part of standard clinical care and should ideally be undertaken soon after time of diagnosis.

To our knowledge, this is the first study to investigate exercise preferences, barriers, and perceived benefits in a relativity large sample of head and neck cancer survivors, and the first in the UK. A limitation of the study was the poor questionnaire response rate of 43%, which is likely somewhat related to the burden of responding to multiple questionnaires in the questionnaire pack. It is also plausible that non-responders were less likely to have been physically active, or interested in participating in an exercise program for head and neck cancer survivors.

## Conclusion

These findings provide exercise preferences to guide exercise program design for head and neck cancer survivors. Exercise barriers specific to head and neck cancer were commonly cited and need addressing to promote exercise uptake and adherence. The need for education on the potential benefits of exercise to promote greater exercise uptake and adherence also was apparent, particularly for those not interested or less interested in participating in an exercise program.
